# The mitochondrial genomes of five spring and groundwater amphipods of the family Crangonyctidae (Crustacea: Amphipoda) from eastern North America

**DOI:** 10.1080/23802359.2021.1926350

**Published:** 2021-05-18

**Authors:** Joseph B. Benito, Megan L. Porter, Matthew L. Niemiller

**Affiliations:** a Department of Biological Sciences, The University of Alabama in Huntsville, Huntsville, AL, USA; b School of Life Sciences, University of Hawai'i at Mānoa, Honolulu, HI, USA

**Keywords:** Crustacean, mitogenome, Amphipoda, Crangonyctidae, *Stygobromus pizzinii*, *Stygobromus allegheniensis*, *Stygobromus tenuis potomacus*, *Bactrurus brachycaudus*, *Crangonyx forbesi*

## Abstract

We sequenced the mitochondrial genomes of one spring-dwelling (*Crangonyx forbesi*) and four groundwater amphipods (*Bactrurus brachycaudus*, *Stygobromus allegheniensis*, *S. pizzinii*, and *S. t. potomacus*) from eastern North America using a shotgun sequencing approach on an Illumina HiSeq 4000 (Illumina, San Diego, CA). All five mitochondrial genomes encoded 13 protein-coding genes, 22 transfer RNAs (tRNAs), and two ribosomal RNAs (rRNAs) representative of subphylum Crustacea. Although the four groundwater species exhibited gene orders nearly identical to the ancestral pancrustacean gene order, the spring-dwelling species, *C. forbesi*, possessed a transposition of the trnH–nad4–nad4l loci downstream after nad6–cytb–trnS2. Moreover, a long nad5 locus, longer rrnL, and rrnS loci, and unconventional start codons distinguished *C. forbesi* from the four groundwater amphipods. Overall, our five amphipod mitogenomes add to the increasing publicly available mitogenome resources for amphipods that are not only valuable for studying the evolutionary relationships of this diverse group of crustaceans but for exploring the evolution of mitochondrial genomes in general.

## Introduction

Amphipods of the family Crangonyctidae are a diverse group of crustaceans, many of which are associated with groundwater-related habitats, such as cave streams, small springs, seeps, hyporheic zones, and wells. This family is particularly diverse in groundwater of the eastern United States with over 100 described species in three genera: *Bactrurus*, *Crangonyx*, and *Stygobromus* (Holsinger 1977, 1978; Zhang 1997; Konemann and Holsinger 2002). Much of this diversity is troglomorphic, lacking eyes, pigment, and frequently with attenuated bodies (Holsinger 1977, 1978). The mitogenomes of several amphipods have been sequenced over the last decade (Krebes and Bastrop 2012; Pons et al. 2014; Stokkan et al. 2015; Aunins et al. 2016; Romanova et al. 2016). For the family Crangonyctidae, however, only three mitogenomes are available and all are for species in the genus *Stygobromus* (Aunins et al. 2016).

To provide better representation for the family Crangonyctidae and provide mitogenomic resources for future phylogenetic studies, we describe herein the complete mitogenomes of four species of groundwater amphipods, including *Stygobromus pizzinii* (Shoemaker 1938), *Stygobromus allegheniensis* (Holsinger 1967), *Stygobromus tenuis potomacus* (Holsinger 1967), and *Bactrurus brachycaudus* (Hubricht and Mackin 1940), as well as a surface spring-dwelling species, *Crangonyx forbesi* (Hubricht and Mackin 1940). Mitogenomic data presented are the first available for the genera *Bactrurus* or *Crangonyx*. Aunins et al. (2016) described the mitogenome of *S. tenuis potomacus* previously, but we present the mitogenome sampled from a different population offering the first intraspecific comparison of amphipod mitogenomes. We also provide a comparative analysis of structure and gene order of other amphipod mitogenomes available.

## Materials and methods

Specimens of *Stygobromus pizzinii* and *S. tenuis potomacus* were collected from Pimmit Run Seepage Spring (38.929432°N; −77.118613°W) (Arlington County, VA), in May 2015. Specimens of *Bactrurus brachycaudus* were collected from Fogelpole Cave (38.198055°N; −90.129722°W) (Monroe County, IL), in October 2014. Specimens of *Stygobromus allegheniensis* were collected from Caskey Spring (35.50319°N; −77.85139°W) (Berkeley County, WV), in September 2013. Specimens of *Crangonyx forbesi* were collected from a unidentified spring (Monroe County, IL), in 2013. All specimens were preserved in 100% ethanol in the field. Specimens and DNA extracts are maintained in the CaveBio collection at The University of Alabama in Huntsville. Whole genomic DNA was isolated using the Qiagen DNA Easy Blood and Tissue kit and libraries were prepared using the Illumina TruSeq DNA Library Prep Kit (Illumina Inc., San Diego, CA). Libraries were then paired-end sequenced (2 × 150 bp) on an Illumina HiSeq 4000 platform at the Vincent J. Coates Genomics Sequencing Laboratory at the University of California, Berkeley (supported by NIH S10 OD018174 Instrumentation Grant).

We assessed the quality of the raw reads using FastQC v0.11.5 (Andrews 2010), and the reads were trimmed and filtered using Trimmomatic v0.33 (Bolger et al. 2014). De-novo assembly was carried out using NOVOPlasty v2.6.3 assembler (Dierckxsens et al. 2017). We then annotated the protein-coding genes, transfer RNAs (tRNAs), and ribosomal RNAs (rRNAs) for each of the five mitogenomes using the mitochondrial genome annotation web server MITOS (Bernt et al. 2013). The annotation of tRNAs were further confirmed using the Mitochondrial tRNA finder MiTFi with Amphipoda models to search specific mitochondrial tRNA (Jühling et al. 2012; Romanova et al. 2020). The locations of start and stop codons of protein coding genes were further confirmed using NCBI ORFfinder (Wheeler et al. 2003) and by visual comparison to other published amphipod mitogenomes. The location of the control region was confirmed by the presence of a large intergenic spacer region with a string of thymines found immediately after rrnS and before trnl. However, the control region was located in a different region within the *C. forbesi* mitogenome. The secondary structures of tRNAs were inferred using MITFI (Jühling et al. 2012), a built-in module in MITOS. We downloaded from GenBank the annotated mitogenomes of 18 related amphipods that occupy the groundwater and spring habitats and three isopods as outgroup for comparative analyses (Table 1). The amino acid sequences of 13 protein-coding genes of the five new mitogenomes, 18 previously published amphipod mitogenomes, and three isopod mitogenomes were aligned using MAFFT version 7 (Katoh and Standley 2013). Poorly aligned regions were eliminated using Gblocks version 0.91b (Castresana 2000). The best partitioning strategy and best-fit evolutionary models for each partition were inferred using PartitionFinder version 2.1.1 (Lanfear et al. 2012). Phylogenetic relationships of the 23 amphipod mitogenomes and three isopod mitogenomes using the concatenated 13 protein-coding gene alignment were determined using Bayesian inference in MrBayes v3.2 (Ronquist et al. 2012). All analyses were conducted using the PhyloSuite v1.1.15 (Zhang et al. 2020).

**Table 1.** List of amphipod mitogenomes, including GenBank accession numbers, taxonomy, and length in bp used for comparative analyses, including new mitogenomes generated in this study (in bold).

GenBank accession	Species	Family	Full length (bp)
KU869712	*Stygobromus tenuis potomacus*	Crangonyctidae	14,915
KU869713	*Stygobromus foliatus*	Crangonyctidae	15,563^a^
**MN175619**	***Bactrurus brachycaudus***	**Crangonyctidae**	**14,661**
**MN175620**	***Stygobromus pizzinii***	**Crangonyctidae**	**15,176**
**MN175621**	***Stygobromus tenuis potomacus***	**Crangonyctidae**	**14,712**
**MN175622**	***Stygobromus allegheniensis***	**Crangonyctidae**	**15,164**
**MN175623**	***Crangonyx forbesi***	**Crangonyctidae**	**15,469**
NC_008412	*Ligia oceanica*^b^	Ligiidae	15,289
NC_013032	*Metacrangonyx longipes*	Metacrangonyctidae	14,113
NC_013976	*Eophreatoicus sp. 14 FK-2009*^b^	Phreatoicidae	14,994
NC_017760	*Gammarus duebeni*	Gammaridae	15,651
NC_019653	*Metacrangonyx repens*	Metacrangonyctidae	14,355
NC_019654	*Metacrangonyx dominicanus*	Metacrangonyctidae	14,543
NC_019655	*Metacrangonyx goulmimensis*	Metacrangonyctidae	14,507
NC_019656	*Metacrangonyx ilvanus*	Metacrangonyctidae	14,770
NC_019657	*Metacrangonyx spinicaudatus*	Metacrangonyctidae	15,037
NC_019658	*Metacrangonyx longicaudus*	Metacrangonyctidae	14,711
NC_019659	*Metacrangonyx panousei*	Metacrangonyctidae	14,478
NC_019660	*Metacrangonyx remyi*	Metacrangonyctidae	14,787
NC_023104	*Eulimnogammarus verrucosus*	Eulimnogammaridae	15,315
NC_024054	*Limnoria quadripunctata*^b^	Limnoriidae	16,515
NC_025564	*Eulimnogammarus vittatus*	Eulimnogammaridae	15,534
NC_030261	*Stygobromus indentatus*	Crangonyctidae	14,638
NC_033360	*Eulimnogammarus cyaneus*	Eulimnogammaridae	14,370
NC_034937	*Gammarus fossarum*	Gammaridae	15,989
NC_037481	*Gammarus roeselii*	Gammaridae	16,073

^a^ Partial mitogenome.

^b^ Isopod mitogenomes.

## Results and discussion

We assembled and annotated the complete mitogenomes of *Stygobromus pizzinii* (15,176 bp, GenBank accession no. MN175620), *Stygobromus tenuis potomacus* (14,712 bp, GenBank accession no. MN175621), *Stygobromus allegheniensis* (15,164 bp, GenBank accession no. MN175622), *Bactrurus brachycaudus* (14,661 bp, GenBank accession no. MN175619), and *Crangonyx forbesi* (15,469 bp, GenBank accession no. MN175623). All mitogenomes contained 13 protein-coding genes, 22 tRNA genes, one small ribosomal RNA (rrnS) gene, and one large ribosomal RNA (rrnL) gene, and a non-coding control region representative of the Kingdom Animalia. In all mitogenomes, the heavy strand encoded a total of 23 genes, whereas the light strand encoded 14 genes. Similar to the ancestral gene order of pancrustaceans (crustaceans and hexapods; Kilpert and Podsiadlowski 2006; Yang and Yang 2008), the light strand encoded the same four protein-coding genes (nad1, nad4, nad4l, and nad5) in all five mitogenomes. AT content of the *Stygobromus* and *Crangonyx* mitogenomes ranged 67.2–69.1%, consistent with the mitogenomes of other amphipods (Aunins et al. 2016; Romanova et al. 2016). However, AT content of the *B. brachycaudus* mitogenome was slightly lower at 63.9%.

Both *C. forbesi* and *S. allegheniensis* had more intergenic spacers (18 and 16, respectively) than *B. brachycaudus*, *S. pizzinii*, or *S. tenuis potomacus*, which all possessed just eight intergenic spacers. These intergenic spacers were found primarily between protein-coding genes, but at times also between tRNA genes. The high number of spacers in *C. forbesi* and *S. allegheniensis* could be a result of gene rearrangement (De Melo Rodovalho et al. 2014). However, additional studies characterizing the mitochondrial genomes of other Crangonyctidae species are needed to better understand the possible association of these intergenic spacers with gene rearrangements.

Gene order in *S. tenuis potomacus*, *S. pizzinii*, *S. allegheniensis*, and *B. brachycaudus* was almost identical to the ancestral pancrustacean gene order (Boore et al. 1998; Pons et al. 2014), except for the transposition of a few tRNA genes. However, *C. forbesi* exhibited quite distinctive gene rearrangements. *Stygobromus pizzinii*, *S. tenuis potomacus*, *S. allegheniensis*, and *B. brachycaudus* shared the conserved gene order of trnF–nad5–trnH–nad4–nad4l with other amphipod mitogenomes. Surprisingly, *C. forbesi* had a transposition of trnH–nad4–nad4l downstream after nad6–cytb–trnS2. The gene order in *C. forbesi* also differed from the ancestral pancrustacean gene order of nad1–trnL1–rrnL–trnV–rrnS with a transposition of trnV upstream between trnM and nad2 and a transposition of nad1 upstream between trnT and trnM. In addition, *S. tenuis potomacus*, *S. pizzinii*, *S. allegheniensis*, and *B. brachycaudus* shared the conserved gene order of trnC–trnY–trnQ; however, *C. forbesi* exhibited a transposition of trnC downstream after trnl–trnG. In addition, *C. forbesi* displayed several other transposed tRNA genes when compared to other amphipods.

ATN start codons, including ATT, ATC, ATG, and ATA, were the most frequently used start codons of most protein-coding genes. However, a few unconventional start codons were also used by protein-coding genes of a few species, including TTG for the nad1 locus in *S. tenuis potomacus*, *S. pizzinii*, *S. allegheniensis*, and *B. brachycaudus*. However, *C. forbesi* used start codon GTG for nad1. Another unconventional start codon included GTG for the cox2 locus in *S. tenuis potomacus* and *S. pizzinii* that has not been observed in other amphipod mitogenomes. In addition, *S. allegheniensis* used start codon GTG for the nad5 locus. Most of the protein-coding genes used TAA or TAG stop codons; however, there were genes that used an incomplete TA– or T– – stop codon. Previous studies have shown that these incomplete stop codons are frequently observed in other amphipods (Pons et al. 2014; Romanova et al. 2016). These incomplete stop codons can be modified into complete stop codons by post-transcriptional polyadenylation (Ojala et al. 1981).

Variation in the length of protein-coding genes and overlap between some adjacent protein-coding genes were observed among the five new crangonyctid mitogenomes and when in comparison with other amphipod mitogenomes. The nad5 gene of *C. forbesi* was 1974 bp in length and was substantially larger than in other amphipods (1665–1719 bp). This length difference is because of additional nucleotides found at the 3′ end of the nad5 gene. Contrastingly, the nad2 gene of *S. allegheniensis* was 895 bp in length and was shorter than nad2 in other species (943–1023 bp). In addition, the nad6 gene of *B. brachycaudus* was 531 bp in length and was longer than in other species (486–507 bp), while the cytb gene was 1098 bp in length and was shorter than in other compared species (1117–1140 bp). The atp6 gene of *S. tenuis potomacus*, *S. pizzinii*, and *S. allegheniensis* overlapped with the adjacent atp8 gene by 41 bp, whereas no overlap was observed in *B. brachycaudus* and *C. forbesi*. An overlap between the atp8 and atp6 genes has been reported in other *Stygobromus* species including *S. tenuis* and *S. indentatus* (Aunins et al. 2016).

All 22 tRNA genes were encoded in the mitogenomes of *S. tenuis potomacus*, *S. pizzinii*, *S. allegheniensis*, *B. brachycaudus*, and *C. forbesi*. A total of 14 tRNA genes were encoded on the heavy strand, and a total of eight tRNA genes were encoded on the light strand in all species. However, *C. forbesi* possessed several transposed tRNAs when compared to other amphipods and the ancestral pancrustacean gene order. The length of the tRNAs ranged from 50 to 66 bp, and most of the tRNAs had ideal cloverleaf secondary structures. However, trnS1 and trnS2 of *S. tenuis potomacus*, *S. pizzinii*, *B. brachycaudus*, and *C. forbesi* were missing the D loop, while trnQ was missing the TΨC loop, which has been observed previously in other *Stygobromus* species (Aunins et al. 2016). In addition, tRNAs of *B. brachycaudus* and *S. allegheniensis* displayed additional unique differences. The tRNAs trnC and trnR of *B. brachycaudus* lacked the D loop and trnQ lacked the acceptor stem in addition to missing the TΨC loop, which was not observed in other species. In contrast, tRNAs trnS1 and trnS2 of *S. allegheniensis* contained the D loop.

Alignment of the large ribosomal RNA (rrnL) gene of all five crangonyctid amphipods revealed the presence of a long sequence (52 bp) overhang on the 5′ end of the rrnL gene of *C. forbesi*. In the crangonyctid amphipod mitogenomes, the rrnL gene was flanked by tRNAs trnL1 and trnV; however, the rrnL gene of *C. forbesi* was immediately located upstream of the small ribosomal RNA (rrnS) and both loci were flanked by tRNAs trnL1 and trnI. The length of the rrnL gene in the other crangonyctid species (1034–1037 bp) was similar to that of other amphipod species.

The small ribosomal RNA (rrnS) gene of all five amphipod species had a relatively conserved 3′ end. Analogous to the rrnL gene, a long sequence (47 bp) overhang was observed on the 5′ end of the rrnS gene in *C. forbesi*. The 3′ end of the rrnS gene in all crangonyctid mitogenomes was followed by a continuous stretch of thymines, which was identified as the 5′ end of the non-coding control region. However, the rrnS gene in *C. forbesi* was immediately followed by tRNA trnl. The control region of *C. forbesi* contained a transposition and was located downstream of the nad1 gene.

A comparison between the two *S. tenuis potomacus* mitogenomes revealed few differences. Their gene order and AT content (69.1%) were identical, and both had equal numbers of intergenic spacers. The unconventional start codon GTG for the cox2 locus found in the *S. tenuis potomacus* (MN175621) mitogenome we sequenced was not observed in the *S. tenuis potomacus* sequenced previously (KU869712; Aunins et al. 2016). The conventional start codon ATC was used instead. In addition, start codons for three other protein-coding loci (cox1, nad3, and nad5) differed in their 3rd position between the two mitogenomes. The cytb locus of *S. tenuis potomacus* (KU869712) used the incomplete stop codon T—while the conventional stop codon TAA was used in *S. tenuis potomacus* (MN175621). In addition, the stop codon for nad3 locus differed in their 3rd position (TAA versus TAG) between the two mitogenomes. Another difference observed was in lengths of the non-coding control region and nad3 locus found. The control region of *S. tenuis potomacus* (KU869712) was 773 bp in length and was substantially larger than in *S. tenuis potomacus* (MN175621, 556 bp). In addition, the nad3 locus of *S. tenuis potomacus* (KU869712) was 381 bp in length and larger than in *S. tenuis potomacus* (MN175621, 354 bp). Apart from these differences, the location, structure, and length of transfer and ribosomal RNAs were nearly identical between the two mitogenomes.

**Figure 1.**
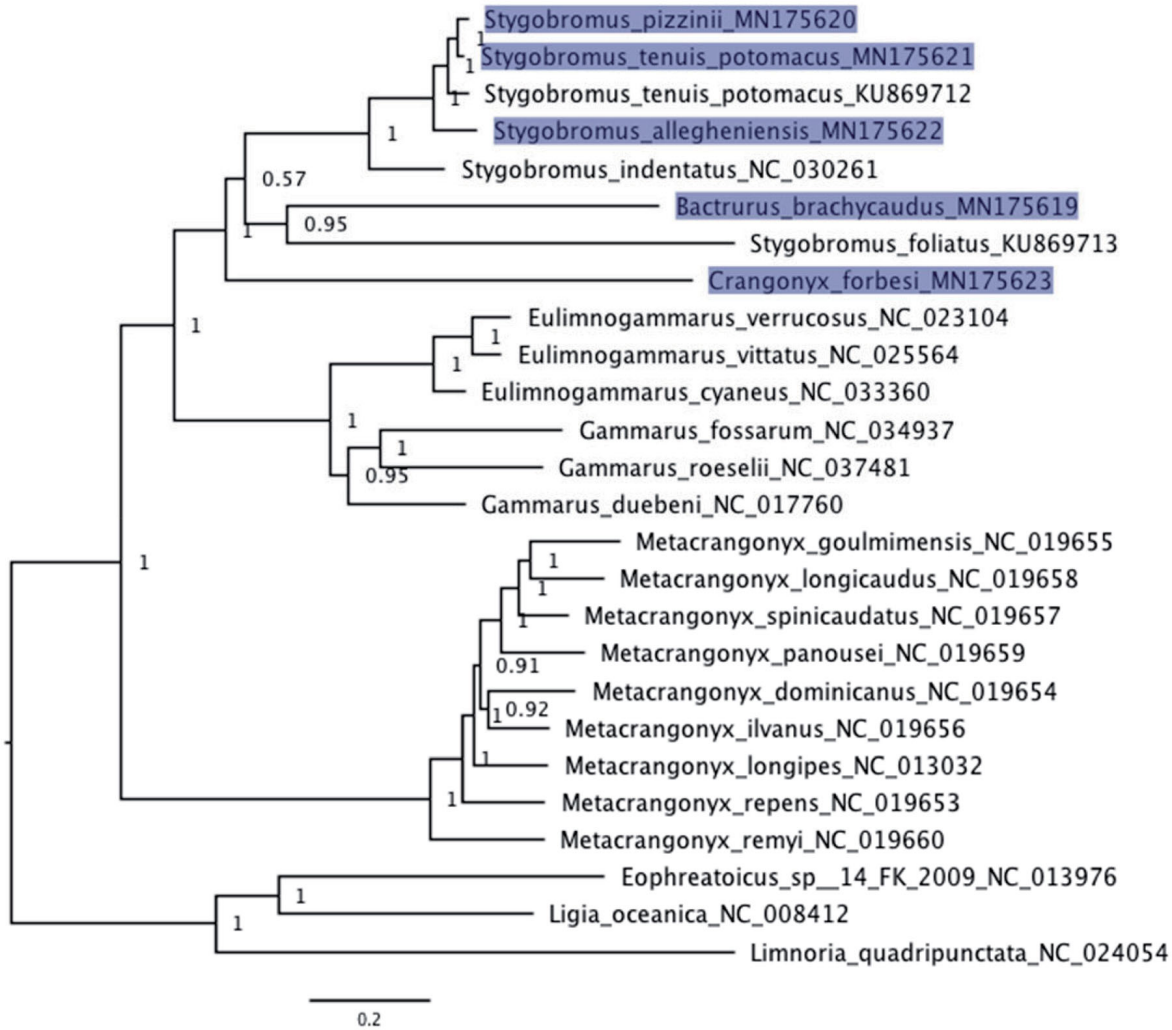
Bayesian phylogeny of aligned protein-coding loci (3169 aa) for five new amphipod mitogenomes (*Stygobromus allegheniensis*, *S. pizzinii*, *S. tenuis potomacus*, *Bactrurus brachycaudus*, and *Crangonyx forbesi*) in addition to 18 additional amphipod mitogenomes available on GenBank. The three isopods *Ligia oceanica*, *Limnoria quadripunctata*, and *Eophreatoicus sp.14 FK-2009* were included as an outgroup to root the phylogeny. New mitogenomes generated in this study are highlighted. GenBank accession numbers were included as suffix next to the species names. Values at nodes represent posterior probabilities.

Bayesian phylogenetic inference of the 13 protein-coding gene alignment (Figure 1) yielded a topology congruent with previous studies of amphipod phylogenetic relationships (Aunins et al. 2016; Romanova et al. 2016; Yang et al. 2017). Members of Crangonyctidae (*Bactrurus*, *Crangonyx*, and *Stygobromus*) form a well-supported clade. However, the genus *Stygobromus* was not monophyletic, as *B. brachycaudus* and *C. forbesi* were nested within *Stygobromus*. *Crangonyx forbesi* was sister to all other crangonyctids, although support for this relationship was lower. The paraphyly of *Stygobromus* also was uncovered in a phylogenetic analysis of the cox1 locus (Niemiller et al. 2018).

## Conclusions

In this study, we have added the complete mitogenomes of four groundwater amphipods – *S. pizzinii*, *S. allegheniensis*, *S. tenuis potomacus*, and *B. brachycaudus* – as well as the complete mitogenome of a spring-dwelling amphipod *C. forbesi* to the growing list of publicly available amphipod mitogenomes. Although all five new mitogenomes exhibited the complete set of 37 genes commonly observed in other amphipods, *C. forbesi* displayed several unique gene arrangements, including the transposition of genes trnH–nad4–nad4l downstream after nad6–cytb–trnS2. This particular gene arrangement deviates significantly from the ancestral pancrustacean gene order and has not been detected in other amphipod species to date. Phylogenetic analysis supports the monophyly of Crangonyctidae but suggests that the genus *Stygobromus* is not a monophyletic group. In general, our contribution of these five additional crangonyctid mitogenomes will be highly valuable for inferring the phylogenetic relationships, biogeography, and trait evolution of amphipods and investigating mitogenome evolution.

## Data Availability

The data that support the findings of this study are openly available in NCBI GenBank at https://www.ncbi.nlm.nih.gov/nuccore/MN175619.1 (*Bactrurus brachycaudus*), https://www.ncbi.nlm.nih.gov/nuccore/MN175620.1 (*Stygobromus pizzinii*), https://www.ncbi.nlm.nih.gov/nuccore/MN175621.1 (*Stygobromus tenuis potomacus*), https://www.ncbi.nlm.nih.gov/nuccore/MN175622.1 (*Stygobromus allegheniensis*), and https://www.ncbi.nlm.nih.gov/nuccore/MN175623.1 (*Crangonyx forbesi*). The sample voucher numbers, related meta-data, and raw sequencing data are openly available in NCBI SRA RunSelector at https://www.ncbi.nlm.nih.gov/Traces/study/?acc=PRJNA657640.

## References

[CIT0001] Aunins AW, Nelms DL, Hobson CS, King TL. 2016. Comparative mitogenomic analyses of three North American stygobiont amphipods of the genus *Stygobromus* (Crustacea: Amphipoda). Mitochondrial DNA Part B. 1(1):560–563. 33473556 10.1080/23802359.2016.1174086 PMC7800481

[CIT0002] Andrews S. 2010. FastQC: a quality control tool for high throughput sequence data. http://www.bioinformatics.babraham.ac.uk/projects/fastqc.

[CIT0003] Bernt M, Donath A, Jühling F, Externbrink F, Florentz C, Fritzsch G, Pütz M, Middendorf M, Stadler PF. 2013. MITOS: improved de novo metazoan mitochondrial genome annotation. Mol Phylogenet Evol. 69:313–319. 22982435 10.1016/j.ympev.2012.08.023

[CIT0004] Bolger AM, Lohse M, Usadel B. 2014. Trimmomatic: a flexible trimmer for Illumina sequence data. Bioinformatics. 30(15):2114–2120. 24695404 10.1093/bioinformatics/btu170 PMC4103590

[CIT0005] Boore JL, Lavrov DV, Brown WM. 1998. Gene translocation links insects and crustaceans. Nature. 392:667–668. 9565028 10.1038/33577

[CIT0006] Castresana J. 2000. Selection of conserved blocks from multiple alignments for their use in phylogenetic analysis. Mol Biol Evol. 17(4):540–552. 10742046 10.1093/oxfordjournals.molbev.a026334

[CIT0007] De Melo Rodovalho C, Lyra ML, Ferro M, Bacci M Jr. 2014. The mitochondrial genome of the leaf-cutter ant *Atta laevigata*: a mitogenome with a large number of intergenic spacers. PLOS One. 9(5):e97117. 24828084 10.1371/journal.pone.0097117 PMC4020775

[CIT0008] Dierckxsens N, Mardulyn P, Smits G. 2017. NOVOPlasty: de novo assembly of organelle genomes from whole genome data. Nucleic Acids Res. 45(4):e18. 28204566 10.1093/nar/gkw955 PMC5389512

[CIT0009] Holsinger JR. 1967. Systematics, speciation, and distribution of the subterranean amphipod genus *Stygonectes* (Gammaridae). Smithsonian Contrib Zool. 259:1–176.

[CIT0010] Holsinger JR. 1977. A review of the systematics of the Holarctic amphipod family Crangonyctidae. Crustaceana Suppl. 4:244–281.

[CIT0011] Holsinger JR. 1978. Systematics of the subterranean amphipod genus *Stygobromus* (Crangonyctidae): part II. Species of the eastern United States. Smithsonian Contrib Zool. 266:1–144.

[CIT0012] Hubricht L, Mackin J. 1940. Descriptions of nine new species of fresh-water Amphipod Crustaceans with notes and new localties for other species. Am Midland Nat. 23(1):187–218.

[CIT0013] Jühling F, Pütz J, Bernt M, Donath A, Middendorf M, Florentz C, Stadler PF. 2012. Improved systematic tRNA gene annotation allows new insights into the evolution of mitochondrial tRNA structures and into the mechanisms of mitochondrial genome rearrangements. Nucleic Acids Res. 40(7):2833–2845. 22139921 10.1093/nar/gkr1131 PMC3326299

[CIT0014] Katoh K, Standley DM. 2013. MAFFT multiple sequence alignment software version 7: improvements in performance and usability. Mol Biol Evol. 30:772–780. 23329690 10.1093/molbev/mst010 PMC3603318

[CIT0015] Kilpert F, Podsiadlowski L. 2006. The complete mitochondrial genome of the common sea slater, *Ligia oceanica* (Crustacea, Isopoda) bears a novel gene order and unusual control region features. BMC Genomics. 7(1):241. 16987408 10.1186/1471-2164-7-241 PMC1590035

[CIT0016] Konemann S, Holsinger JR. 2002. Systematics of the North American subterranean amphipod genus *Bactrurus* (Crangonyctidae). Beaufortia. 51:1–56.

[CIT0017] Krebes L, Bastrop R. 2012. The mitogenome of *Gammarus duebeni* (Crustacea Amphipoda): a new gene order and non-neutral sequence evolution of tandem repeats in the control region. Comp Biochem Physiol D Genomics Proteomics. 7(2):201–211. 22437035 10.1016/j.cbd.2012.02.004

[CIT0018] Lanfear R, Calcott B, Ho SY, Guindon S. 2012. PartitionFinder: combined selection of partitioning schemes and substitution models for phylogenetic analyses. Mol Biol Evol. 29(6):1695–1701. 22319168 10.1093/molbev/mss020

[CIT0019] Niemiller ML, Porter ML, Keany J, Gilbert H, Fong DW, Culver DC, Hobson CS, Kendall KD, Davis MA, Taylor SJ. 2018. Evaluation of eDNA for groundwater invertebrate detection and monitoring: a case study with endangered *Stygobromus* (Amphipoda: Crangonyctidae). Conserv Genet Resour. 10(2):247–257.

[CIT0020] Ojala D, Montoya J, Attardi G. 1981. tRNA punctuation model of RNA processing in human mitochondria. Nature. 290:470–474. 7219536 10.1038/290470a0

[CIT0021] Pons J, Bauzà-Ribot MM, Jaume D, Juan C. 2014. Next-generation sequencing, phylogenetic signal and comparative mitogenomic analyses in *Metacrangonyctidae* (Amphipoda: Crustacea). BMC Genomics. 15(1):566. 24997985 10.1186/1471-2164-15-566 PMC4112215

[CIT0022] Romanova EV, Aleoshin VV, Kamaltynov RM, Mikhailov KV, Logacheva MD, Sirotinina EA, Gornov AY, Anikin AS, Sherbakov DY. 2016. Evolution of mitochondrial genomes in Baikalian amphipods. BMC Genomics. 17(14):1016. 28105939 10.1186/s12864-016-3357-z PMC5249044

[CIT0023] Romanova EV, Bukin YS, Mikhailov KV, Logacheva MD, Aleoshin VV, Sherbakov DY. 2020. Hidden cases of tRNA gene duplication and remolding in mitochondrial genomes of amphipods. Mol Phylogenet Evol. 144:106710. 31846708 10.1016/j.ympev.2019.106710

[CIT0024] Ronquist F, Teslenko M, van der Mark P, Ayres DL, Darling A, Höhna S, Larget B, Liu L, Suchard MA, Huelsenbeck JP. 2012. MrBayes 3.2: efficient Bayesian phylogenetic inference and model choice across a large model space. Syst Biol. 61:539–542. 22357727 10.1093/sysbio/sys029 PMC3329765

[CIT0025] Shoemaker CR. 1938. A new species of fresh-water amphipod of the genus *Synpleonia*, with remarks on related genera. Proc Biol Soc Washington. 51:137–142.

[CIT0026] Stokkan M, Jurado-Rivera J, Carlos J, Damià J, Pons J. 2015. Mitochondrial genome rearrangements at low taxonomic levels: three distinct mitogenome orders in the genus *Pseudoniphargus*. Mitochondrial DNA. 27:3579–3589. 26329687 10.3109/19401736.2015.1079821

[CIT0027] Wheeler DL, Church DM, Federhen S, Lash AE, Madden TL, Pontius JU, Schuler GD, Schriml LM, Sequeira E, Tatusova TA, et al. 2003. Database resources of the National Center for Biotechnology. Nucleic Acids Res. 31(1):28–33. 12519941 10.1093/nar/gkg033 PMC165480

[CIT0028] Yang HM, Song JH, Kim MS, Min GS. 2017. The complete mitochondrial genomes of two talitrid amphipods, *Platorchestia japonica* and *P. parapacifica* (Crustacea, Amphipoda). Mitochondrial DNA Part B. 2(2):757–758. 33473971 10.1080/23802359.2017.1398606 PMC7799624

[CIT0029] Yang JS, Yang WJ. 2008. The complete mitochondrial genome sequence of the hydrothermal vent galatheid crab *Shinkaia crosnieri* (Crustacea: Decapoda: Anomura): a novel arrangement and incomplete tRNA suite. BMC Genomics. 9(1):257. 18510775 10.1186/1471-2164-9-257 PMC2442616

[CIT0030] Zhang D, Gao F, Li WX, Jakovlić I, Zou H, Zhang J, Wang GT. 2020. PhyloSuite: an integrated and scalable desktop platform for streamlined molecular sequence data management and evolutionary phylogenetics studies. Mol Ecol Resour. 20(1):348–355. 31599058 10.1111/1755-0998.13096

[CIT0031] Zhang J. 1997. Systematics of the freshwater Amphipod genus *Crangonyx* (Crangonyctidae) in North America [Doctor of Philosophy (PhD), dissertation]. Biological Sciences, Old Dominion University.

